# Epiglottopexy Is a Treatment of Choice for Obstructive Sleep Apnea Caused by a Collapsing Epiglottis

**DOI:** 10.3390/life12091378

**Published:** 2022-09-05

**Authors:** Michaela Masárová, Martin Formánek, Ondřej Jor, Vilém Novák, Adéla Vrtková, Petr Matoušek, Pavel Komínek, Karol Zeleník

**Affiliations:** 1 Department of Otorhinolaryngology and Head and Neck Surgery, University Hospital Ostrava, 708 52 Ostrava, Czech Republic; 2 Faculty of Medicine, University of Ostrava, 708 52 Ostrava, Czech Republic; 3 Department of Anesthesiology, Resuscitation and Intensive Medicine, University Hospital Ostrava, 708 52 Ostrava, Czech Republic; 4 Department of Pediatric Neurology, University Hospital Ostrava, 708 52 Ostrava, Czech Republic; 5 Department of Applied Mathematics, Faculty of Electrical Engineering and Computer Science, VSB-Technical University of Ostrava, 708 52 Ostrava, Czech Republic

**Keywords:** obstructive sleep apnea, drug-induced sleep endoscopy, epiglottopexy, positive airway pressure

## Abstract

Drug-induced sleep endoscopy (DISE) reveals epiglottic collapse to be a frequent cause of obstructive sleep apnea (OSA) and intolerance of positive airway pressure (PAP). These patients require different management. This prospective study aimed to compare transoral laser epiglottopexy outcomes in patients with OSA caused by epiglottic collapse with the patients’ previous PAP outcomes. Fifteen consecutive adult patients with OSA and epiglottic collapse during DISE were included; ten were analyzed. Before inclusion, PAP was indicated and ineffective in six patients, one of whom underwent unsuccessful uvulopalatopharyngoplasty. PAP was performed during DISE in all patients before epiglottopexy and was uniformly ineffective. ENT control was performed at 1 week and 1 month, and control limited polygraphy to 6 months after surgery. The apnea–hypopnea index (AHI) and Epworth Sleepiness Scale (ESS) were significantly improved (*p* < 0.001 and *p* = 0.003, respectively) in all patients after epiglottopexy. Surgery was successful in 9/10 patients; the remaining patient had a significantly decreased AHI and could finally tolerate PAP. Transoral laser epiglottopexy is used to treat OSA in patients with epiglottic collapse. Unlike other methods, it significantly reduces both AHI and ESS and should be considered for these patients. An active search for OSA patients with epiglottic collapse is recommended to prevent treatment failure.

## 1. Introduction

The role of the epiglottis in the development of obstructive sleep apnea (OSA) in adults has been known for many years [1]. The most common type of epiglottic pathology is “closing door” epiglottis, when the epiglottis collapses on the posterior wall of the hypopharynx during inspiration [2].

In the past, the prevalence of epiglottic collapse evaluated by clinical examination was estimated to be 12% in OSA patients [3]. In recent years, however, drug-induced sleep endoscopy (DISE) has shown that the epiglottis is a much more common cause of OSA [4].

**Figure 1:**
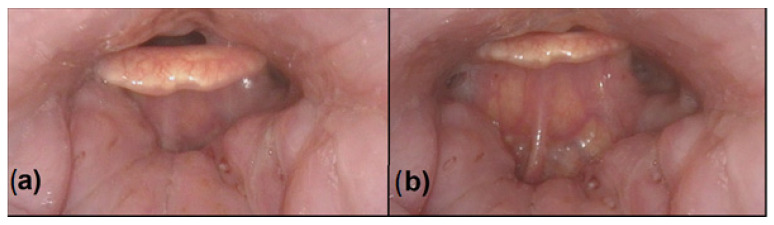
DISE with simultaneous PAP, endoscopic view. (**a**) Anterior–posterior obstruction of the epiglottis as a cause of upper-airway obstruction, without PAP. (**b**) PAP failure: the pressure is 18 hPa and aggravates the upper-airway obstruction by pushing the epiglottis further down into the laryngeal inlet.

Currently, there is a lack of good understanding about the relationship between epiglottic collapse and its most effective treatment. The treatment of OSA with positive airway pressure (PAP) is considered the “gold standard” [5,6]. However, in cases of epiglottic collapse, several studies have suggested that PAP may rather aggravate airway obstruction by pushing the epiglottis further down into the laryngeal inlet (Figure 1) [1]. Thus, these patients could require different management and it seems logical for surgery to play a crucial role regardless of the severity of OSA.

Currently, there are insufficient data regarding the effect of epiglottis intervention [2,7,8].

Therefore, the aim of this prospective study was to evaluate the treatment outcomes of transoral laser epiglottopexy in patients with OSA at different severities caused by epiglottic collapse and to compare them with the outcomes of pre- and perioperative positive airway pressure treatments in these patients.

## 2. Materials and Methods

This prospective study was performed with the consent of the Ethics Committee of the University Hospital Ostrava and performed in accordance with the Declaration of Helsinki and the requirements of good clinical practice. Written informed consent was obtained from each patient before any procedure was initiated.

### 2.1. Study Design

This study was performed at the tertiary referral center.

### 2.2. Inclusion Criteria

Adult patients with OSA confirmed by limited polygraphy or polysomnography with an AHI ≥ 5 episodes/h and epiglottic collapse during DISE.

### 2.3. Exclusion Criteria

Major comorbidities representing excessive risk for general anesthesia, craniofacial malformations, neurological pathologies, patients who did not want surgery or did not agree to their inclusion in the study, and patients in need of multilevel surgery (epiglottopexy and soft palate or oropharyngeal surgery).

### 2.4. Clinical Evaluation

Patients were evaluated by obtaining their comprehensive history covering sleep habits and disturbances: shift work, sleep duration, sleep quality, lack of sleep, daily sleepiness, and snoring. Excessive daytime sleepiness was estimated by the ESS. Each patient’s body mass index (BMI) was also recorded.

Clinical evaluation included a complete head and neck examination that evaluated the presence of soft palatal webbing, the presence of an elongated uvula, and the size of the tonsils according to Friedmann. The relation of the base of the tongue to the soft palate was evaluated using the Mallampati classification.

The upper airways and digestive tract were examined using a flexible videoendoscope with a diameter of 3.5 mm (Olympus, Tokyo, Japan) in each patient. Laterolateral narrowing of the oropharynx, narrowing of the base of the tongue, and the presence of pathology in the epiglottis were assessed according to Kezirian [9].

### 2.5. Drug-Induced Sleep Endoscopy

**Figure 2:**
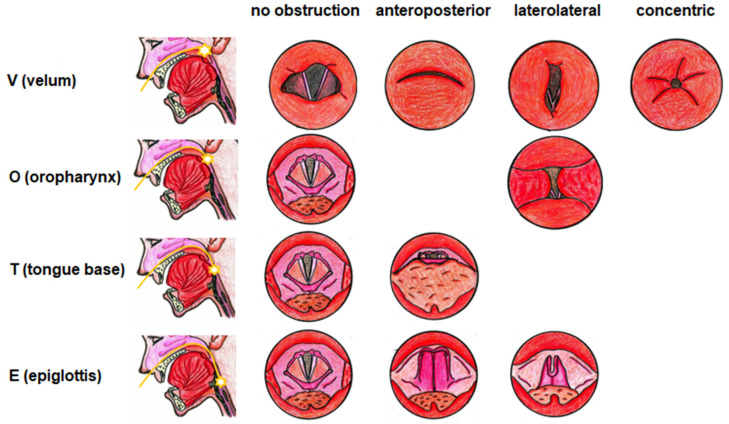
The VOTE classification according to Kezirian (2011). Obstruction in four upper-airway areas is evaluated: the area of the soft palate, the lateral pharynx walls and the tonsils, the tongue base, and the epiglottis. In each of these locations, the degree of obstruction (0, no obstruction; 1, partial obstruction; 2, complete obstruction) and obstruction configuration (anteroposterior, circular, laterolateral) are evaluated [7].

Sleep endoscopy was performed by an experienced otorhinolaryngologist and anesthesiologist in the operating room. After intramuscular administration of 5 mg of Dormicum (midazolam) and 0.5 mg of atropine 30 min before the examination, the patient in supine position without neck extension was induced to sleep with intravenous propofol (a 1 mg/kg bolus at the beginning, then 20–30 mg every 3–5 min). The depth of anesthesia was measured by the bispectral index throughout the examination and maintained in the range of 50–70. Vital signs were monitored and included blood pressure, heart rate, electrocardiogram, oxygen saturation, and respiratory rate. Sleep endoscopy was performed using a flexible videoendoscope with a diameter of 3.5 mm (Olympus, Tokyo, Japan), and the length of the examination was 15–20 min. The Kezirian VOTE classification was used to evaluate the results (Figure 2) [9].

### 2.6. Positive Airway Pressure Titration during the Sleep Endoscopy

The BiPAP A40 (Philips Respironics, Murrysville, PA, USA) in PAP mode was used for titration. PAP titration was performed immediately after DISE with the patient in supine position. An overpressure ventilation mask (Respironics PerforMax Full-face mask, Philips Respironics, Murrysville, PA, USA) of appropriate size was applied to the patient’s face. A special connecting valve (Philips Respironics, Murrysville, PA, USA) was inserted between the mask and the device hose, through which a flexible endoscope was inserted into the nose and nasopharynx. The mask was subsequently fixed with straps.

Sleep endoscopy was then performed under overpressure ventilation. The start of the examination was at a pressure of 6 hPa. Gradually, the PAP was elevated (always after at least 30 s) in the range of 6, 8, 10, 12, 14, and 18 hPa. At each pressure tested, an evaluation was performed by two physicians independently. The efficiency of overpressure ventilation was assessed visually, and at the same time the values of blood oxygen saturation measured on a finger of the upper limb were monitored.

The Kezirian VOTE classification was used for evaluation at each PAP tested to observe the effect of increasing pressure on the obstruction of the monitored localities.

### 2.7. Surgical Technique

In patients with an epiglottic collapse during sleep endoscopy, transoral laser epiglottopexy was subsequently performed under general anesthesia using an operating microscope (Zeiss, Oberkochen, Germany). The patient lay in supine position and their superior teeth were protected by a silicone protector. The base of the tongue and the entire epiglottic vallecula and epiglottis were exposed by a laryngoscope.

**Figure 3:**
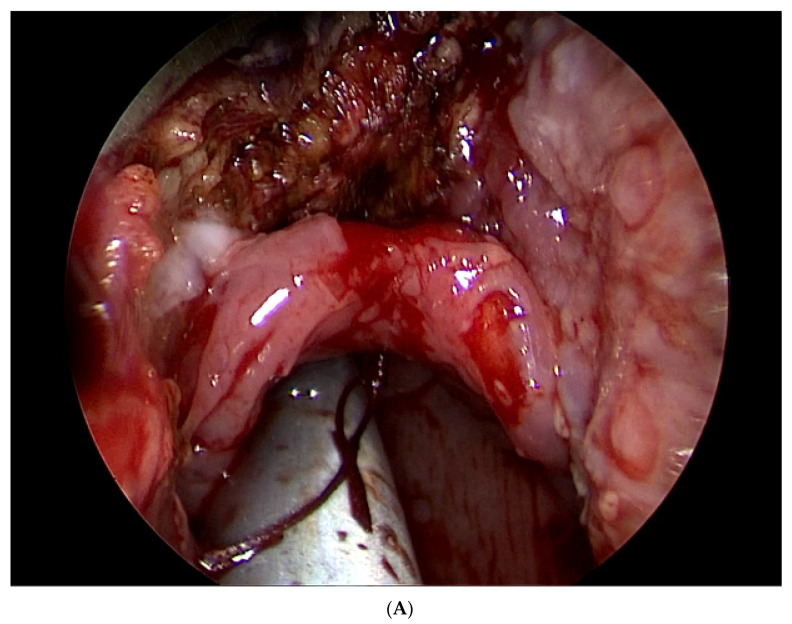

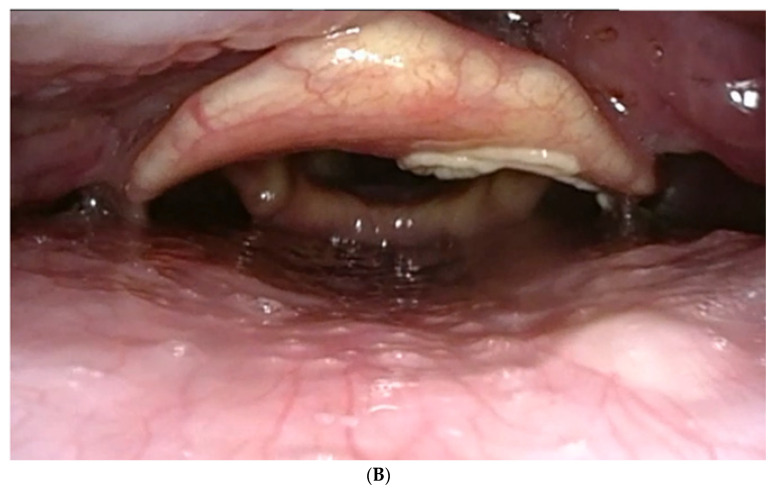
The principle of epiglottopexy. (**A**) Endoscopic peroperative view. (**B**) Follow-up videoendoscopic examination 1 month after surgery.

Removal of the mucosa at the base of the tongue, epiglottic vallecula, and the lingual surface of the epiglottis was performed using a Thulium laser (Revolix, Getz Healthcare, Singapore). The laser was set at 8–10 watts of delivered power. A rim of 5 mm of healthy mucosa was left intact along the entire lingual surface of the epiglottis. After de-epithelialization of the mucosa, the epiglottis was fixed to the base of the tongue with two absorbable stitches (Figure 3A).

Moderate perioperative bleeding was treated with cauterization. Due to the laser’s ability to coagulate vessels, it was usually not necessary to deal with major perioperative bleeding.

### 2.8. Postoperative Care

Antibiotics were administered intravenously for 24 h, and after that orally until the 7th postoperative day. Analgesics were also recommended for 1 week. If the patient had no problems with swallowing, they were allowed to resume oral intake including liquids from the first postoperative day.

### 2.9. Follow-Up

ENT control with a flexible videoendoscope (Olympus, Tokyo, Japan) was performed 1 week and 1 month after the surgery (Figure 3B). Control polysomnography or limited polygraphy was performed 6 months after the surgery. The presence of complications during follow-up was monitored.

### 2.10. Statistical Analysis

The primary analysis was performed using standard tools of exploratory data analysis. For the description of numerical variables, the mean or the median was used. Categorical variables were presented with absolute and/or relative frequencies. The success of surgery and healing were evaluated according to Sher’s criteria [10,11]. Previous therapy was chosen as a control for each patient. Statistical analysis was performed using R software (version 3.6.0; R Core Team, R Foundation for Statistical Computing, Vienna, Austria). The one-tailed Wilcoxon signed-rank test was used with a 5% significance level.

## 3. Results

Altogether, 15 consecutive patients were included in this study. Four patients had to be excluded: three patients did not want surgery and were treated with a mandibular advancement with partial effect, and one patient did not come for surgery. Surgery was performed on 11 patients. One patient was lost to follow-up and had to be excluded from analysis.

A total of 10 patients (eight males and two females) were analyzed. The mean age of analyzed patients was 43.7 years. The patients had varying degrees of nonpositional OSA (mean AHI: 25.9; mean ESS: 15.6), and their mean BMI was 28.6. There were no cases of head and neck cancer or multiple system atrophy in these patients. Before their inclusion in the study, positive airway pressure treatment was primarily indicated as the first-line treatment in six patients and was not effective in any of them according to AHI and ESS. In one patient, uvulopalatopharyngoplasty was primarily performed in another hospital, also with no effect on OSA (Table 1).

According to the awake examination findings, including the use of a flexible endoscope, none of the patients had any epiglottic pathology. Positive airway pressure was not effective during the DISE in any patient, even at the strongest pressure tested, resulting in PAP treatment failure in all patients if it had been indicated, as was confirmed in those six patients who underwent preoperative positive airway pressure treatment. In all cases, positive airway pressure only aggravated the airway obstruction by pushing the epiglottis further down into the laryngeal inlet.

**Figure 4:**
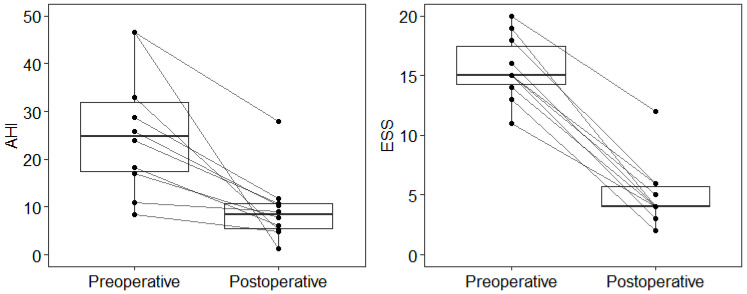
Visualization of postoperative AHI and ESS reduction (paired boxplots). AHI, apnea–hypopnea index; ESS, Epworth sleepiness scale.

Postoperative AHI and ESS were significantly improved in all patients 6 months after epiglottopexy (Table 2; Figure 4). In total, 9/10 (90%) patients did not need further treatment after surgery, and quality of life was significantly improved. Therefore, we assume that the treatment was successful for them, which was also confirmed by Sher’s criteria. There was an almost 40% decrease in AHI (from 46.5 to 28) in the one remaining patient with very severe OSA, who had not tolerated previous PAP treatment. Postoperatively, the patient could tolerate positive airway pressure treatment.

Postoperative bleeding from the base of the tongue was noted in one patient 1 week after surgery, and a revision was performed under general anaesthesia. No other perioperative or postoperative complication was observed. No patient had postoperative swallowing problems. All patients started a liquid diet on the first postoperative day. A soft non-irritating diet was started one week after surgery. All patients were transitioned to a solid diet without any problems one month after surgery.

## 4. Discussion

The morphology of upper-airway structures plays a major role in the pathogenesis of OSA [7,12]. In recent years, DISE has gained popularity in identifying specific anatomical locations and patterns of obstruction. It allows examination of the dynamic status of upper airways during sleep and has become an important part of the armamentarium of surgeons to evaluate their OSA patients [12]. Specifically, it has a strength in identifying epiglottic collapse, which is not easily predictable without sedation [3,4,13].

In the past, the prevalence of epiglottic collapse was estimated to be around 12% in adult OSA patients. Later, and based on DISE, a significantly higher percentage of adult patients with OSA were found to exhibit epiglottic collapse [2,4]. Fernandez-Julian et al. compared surgical recommendations in 162 patients and found that the epiglottis was involved in the obstruction of 36.4% of patients according to DISE, but of only 24.1–28.4% according to awake examination [14]. Koutsourelakis et al. reported 49 OSA patients who were evaluated during DISE before upper-airway surgery and found 36 patients (73.5%) with some degree of epiglottic collapse [15]. Thus, epiglottic collapse seems to play an important role in airway obstruction in patients with OSA [14,15].

The treatment of OSA with positive airway pressure is currently considered as the “gold standard” for many patients [5,6,16]. Although PAP has high efficacy, the effectiveness of the therapy is limited, as demonstrated by 46–83% of patients being non-adherent to therapy (defined as >4 h of use per night) [16]. These patients very often lose interest in further solutions and hope for their healing or are usually offered another possible therapy that consists mainly of uvulopalatopharyngoplasty with or without surgery on the base of the tongue [9,17]. However, this type of surgery is very rarely successful in these patients. Therefore, these patients are forced to take the considerable risks associated with general anesthesia and uvulopalatopharyngoplasty and, ultimately, to live with their OSA. Velopharyngeal OSA surgery has an increased risk of postoperative complications, such as bleeding (sometimes life-threatening), foreign body sensation, dry throat, globus sensation, problems with phlegm, and velopharyngeal insufficiency [17]. Only single-site case series provide current estimates of the incidence of the perioperative complications of UPPP, with a pooled crude serious complication rate of 3.5% and a crude mortality rate of 0.4% [18]. Haavisto et al. reported that as late as 1 year after surgery, 57% of patients had some kind of problem in relation to the operation, the most common complaint being velopharyngeal insufficiency (24%) [19].

According to the literature, a collapsing epiglottis has been found in 15–31.4% of adult patients with OSA who did not tolerate positive airway pressure therapy or in whom positive airway pressure treatment was ineffective [1,3,4,6]. In the case of epiglottic collapse, several studies have shown that rising overpressure of positive airway pressure therapy only pushes the epiglottis onto the back wall of the pharynx more and thus makes the obstruction worse [2,11,20]. Torre et al. states that PAP does not resolve primary epiglottis collapse and too high pressures are required to open it. Furthermore, PAP has been found to be particularly ineffective in cases of latero-lateral epiglottis obstruction. In the case of the latero-lateral epiglottis obstruction the problem persists even at pressures higher than 15.0 hPa [2].

It can be stated that positive airway pressure therapy is not an ideal choice for patients with epiglottic collapse. Positional therapy or mandibular advancement seems to have slightly more promising results. It has been shown that both methods affect epiglottic collapse relatively positively, but only in patients with mild OSA. In patients with a higher AHI these methods often fail and additional treatment is required [21,22,23]. Kent et al. analyzed 35 consecutively screened adult patients with OSA with positive airway pressure therapy intolerance and incomplete response to oral appliance therapy and found 20% of them to have epiglottic collapse [21]. In addition, both treatment modalities (positional therapy and mandibular advancement) require high compliance and do not solve their OSA definitively [3,24]. Less compliant patients are thus at risk of not being properly treated and exposed to all OSA complications.

It is interesting that patients with epiglottis collapse seem to be thinner than standard patients with OSA and their AHI does not correlate with their BMI [25]. Likewise, epiglottis collapse is not usually related to craniofacial deformities, which can also predispose patients to OSA [17,25]. Patients with epiglottic collapse are often young and slim with a different degree of OSA, in our experience. Therefore, especially with these patients, it is very important to think about the possibility of a collapsing epiglottis [2,3].

Based on our results, transoral laser epiglottopexy seems to be an ideal therapeutic option for these patients. There are multiple choices for epiglottic surgery including epiglottidectomy (total, partial, and V-shaped) or transoral epiglottopexy [4,13,26,27]. However, transoral epiglottopexy (or glossoepiglottopexy) is the most gentle surgical technique in terms of impairing the fundamental functions of the epiglottis, complications, and postoperative morbidity [2,3,9,26,27,28,29,30].

Generally, many surgical approaches and techniques in this region are significantly invasive and associated with a high rate of complications, such as bleeding, edema, persistent dysphagia, dysgeusia, etc. However, new technologies (diathermy, CO_2_ laser, thulium laser, and coblation) have allowed the introduction of innovative surgical techniques that are relatively safe [2,3,9].

The functions of the epiglottis must be taken into account. The epiglottis is involved in preventing food aspirations in two ways. It works as a mechanical closure of the laryngeal aditus during swallowing. Its sensitive receptors, distributed over its surface, participate in swallowing as well. In an effort to prevent unwanted complications such as aspirations, it is important from a technical point of view to leave a 3–4 mm rim of healthy mucosa along the entire profile of the epiglottis [3,4,27].

However, to date, no serious complications associated with epiglottopexy have been reported in the literature. In addition, it is a reversible method in case of unexpected complications [3,9]

The most risky procedure is an intervention at the base of the tongue (risking injury to the lingual artery with subsequent perioperative or postoperative bleeding). Therefore, it is very important not to reduce the base of the tongue or expose the mucosa too laterally, but to adhere strictly to the area around the midline [3,8,18,30].

Although our results are limited by the small number of patients, it can be stated that correctly applied epiglottopexy seems to be a very effective first-stage treatment for OSA caused by epiglottic collapse, with minimal risk of complications. What is more, epiglottopexy can significantly improve the tolerance of PAP treatment in some cases and prevent serious complications associated with other upper-airway surgeries (such as tonsillectomy, uvulopalatopharyngoplasty, etc.) that incorrectly diagnosed patients undergo unnecessarily [4,19,30]. A collapsing epiglottis is one of the very frequent reasons for PAP intolerance and cannot be properly evaluated during clinical examination with endoscopy. Thus, DISE should be considered in such cases.

## 5. Conclusions

A collapsing epiglottis is a relatively common cause of OSA and one of the reasons for positive airway pressure intolerance. Since it cannot be detected while awake, DISE should be recommended in these cases. Transoral laser epiglottopexy is an elegant technique that significantly ameliorates the severity of OSA, or in some cases even cures OSA in patients with epiglottic collapse. Unlike other methods, it significantly reduces both AHI and ESS and should be the method of choice for these patients. Despite the intervention in the larynx, it provides stable support to the epiglottis without affecting its function during swallowing. Transoral laser epiglottopexy is thus a safe surgical technique. It is essential to actively seek out patients with epiglottic collapse among other patients with OSA to prevent treatment failure and lessen the risk of potential complications.

## Figures and Tables

**Table 1:** Characteristics of the analyzed patients.

Patient	Age (Years)	Preoperative BMI	Preoperative AHI	Preoperative ESS	Preinclusion Failed Therapy	Awake Examination Findings (Obstruction)	DISE Findings (Obstruction)	Opening PAP Pressure (hPa)	Postoperative BMI	Postoperative AHI	Postoperative ESS
1	22	25.48	46.5	15	PAP	Tonsils	Epiglottis	>18	25.15	1.3	5
2	50	30.88	33	16	PAP UPPP	Tongue base	Epiglottis Tongue base	>18	31.25	5.3	4
3	44	29.32	8.3	11	-	Soft palate Tongue base	Epiglottis	>18	28.76	4.8	4
4	58	31.1	46.5	20	PAP	Soft palate Tongue base	Epiglottis, Tongue base	>18	31.15	28	12
5	49	26.59	18.3	18	-	Soft palate Tongue base	Epiglottis Tongue base	>18	25.48	6.1	6
6	48	27.65	28.83	19	PAP	Soft palate Tongue base	Epiglottis, Tongue base	>18	28.88	11.7	4
7	32	28.47	25.74	15	PAP	Soft palate Tongue base	Epiglottis Tongue base	>18	29.32	10.2	3
8	41	26.2	23.99	15	PAP	Soft palate	Epiglottis Tongue base	>18	29.7	10.8	6
9	38	32	11	13	-	Soft palate Tongue base	Epiglottis	>18	31.67	9	2
10	55	28	17.1	14	-	Soft palate Tongue base	Epiglottis Tongue base	>18	26.54	7.7	4

BMI, body mass index; AHI, apnea–hypopnea index; ESS, Epworth sleepiness scale; PAP, positive airway pressure.

**Table 2:** Analysis of AHI and ESS postoperative reduction.

	Median (Min; Max)			
	Preoperative	Postoperative	Difference	p *
**AHI**	24.9 (8.3; 46.5)	8.4 (1.3; 28.0)	14.4 (2.0; 45.2)	<0.001
**ESS**	15 (11; 20)	4 (2; 12)	11 (7; 15)	0.003

Difference = Preoperative–Postoperative; AHI, apnea–hypopnea index; ESS, Epworth sleepiness scale. * The *p*-value of the one-tailed Wilcoxon signed-rank test.

## Data Availability

All available data presented.
